# Physiologically-Based Pharmacokinetic-Pharmacodynamics Model Characterizing CYP2C19 Polymorphisms to Predict Clopidogrel Pharmacokinetics and Its Anti-Platelet Aggregation Effect Following Oral Administration to Coronary Artery Disease Patients With or Without Diabetes

**DOI:** 10.3389/fphar.2020.593982

**Published:** 2020-12-17

**Authors:** Ru-jun Xu, Wei-min Kong, Xiao-fei An, Jian-jun Zou, Li Liu, Xiao-dong Liu

**Affiliations:** ^1^ Center of Pharmacokinetics and Metabolism, College of Pharmacy, China Pharmaceutical University, Nanjing, China; ^2^ Department of Endocrinology, Affiliated Hospital of Nanjing University of Chinese Medicine, Jiangsu Province Hospital of Chinse Medicine, Nanjing, China; ^3^ Department of Clinical Pharmacology, Nanjing First Hospital, Nanjing Medical University, Nanjing, China; ^4^ School of Basic Medicine and Clinical Pharmacy, China Pharmaceutical University, Nanjing, China

**Keywords:** clopidogrel, PBPK-PD model, coronary artery disease, diabetes mellitus, CYP2C19 polymorphism, carboxylesterase 1 activity

## Abstract

**Background and Objective:** Clopidogrel (CLOP) is commonly used in coronary artery disease (CAD) patients with or without diabetes (DM), but these patients often suffer CLOP resistance, especially those with diabetes. This study was aimed to develop a physiologically-based pharmacokinetic-pharmacodynamic (PBPK-PD) model to describe the pharmacokinetics and pharmacodynamics of clopidogrel active metabolite (CLOP-AM) in CAD patients with or without DM.

**Methods:** The PBPK-PD model was first established and validated in healthy subjects and then in CAD patients with or without DM. The influences of CYP2C19, CYP2C9, CYP3A4, carboxylesterase 1 (CES1), gastrointestinal transit rates (*K*_t,i_) and platelets response to CLOP-AM (*k*_irre_) on predicted pharmacokinetics and pharmacodynamics were investigated, followed with their individual and integrated effects on CLOP-AM pharmacokinetics due to changes in DM status.

**Results:** Most predictions fell within 0.5–2.0 folds of observations, indicating successful predictions. Sensitivity analysis showed that contributions of interested factors to pharmacodynamics were CES1> *k*_irre_> *K*_t,i_> CYP2C19 > CYP3A4> CYP2C9. Mimicked analysis showed that the decreased exposure of CLOP-AM by DM was mainly attributed to increased CES1 activity, followed by decreased CYP2C19 activity.

**Conclusion:** The pharmacokinetics and pharmacodynamics of CLOP-AM were successfully predicted using the developed PBPK-PD model. Clopidogrel resistance by DM was the integrated effects of altered *K*_t,i_, CYP2C19, CYP3A4, CES1 and *k*_irre_.

## Introduction

Clopidogrel (CLOP) is a thienopyridine antiplatelet agent used widely in the prevention of cardiovascular events in coronary artery disease (CAD) patients. CLOP is a prodrug, which is converted into its active metabolite (CLOP-AM) to exhibit the anti-platelet effect (Savi et al., 1992). After oral administration, 85–90% of the absorbed CLOP is converted into inactive carboxylic acid metabolite by carboxylesterase 1 (CES1) and only 10∼15% of absorbed CLOP is metabolized to intermediate metabolite 2-oxo-clopidogrel (2-oxo-CLOP) via CYP1A2, CYP2B6 and CYP2C19 (Jiang et al., 2015). Then, about 50% of 2-oxo-CLOP is hydrolyzed by CES1 to an inactive form and remaining ∼50% of 2-oxo-CLOP is metabolized to CLOP-AM by CYP2B6, CYP2C9, CYP2C19 and CYP3A4 (Djebli et al., 2015). Finally, only 2% of the administered CLOP dose is converted into CLOP-AM and reaches the systemic circulation (Jiang et al., 2015). The CLOP-AM is further hydrolyzed by CES1. Once CLOP-AM is formed, it will irreversibly bind to adenosine diphosphate (ADP) receptor P2Y12 on the surface of platelets, inhibiting the ADP-induced platelet aggregation (Jiang et al., 2015).

The roles of CYP450s in the formation of CLOP-AM have been further demonstrated, especially CYP2C19, which contributes to about 50% of CLOP-AM formation (Jiang et al., 2015), showing a more important role in CLOP’s bioactivation than other CYP450s. Clinical evidence has demonstrated that CYP2C19 polymorphisms are often associated with CLOP resistance (Jiang et al., 2015). Pharmacokinetic-pharmacodynamic (PK-PD) investigations have revealed that subjects carrying loss-function alleles (CYP2C19*2 or CYP2C19*3) have significantly lower systemic exposure of CLOP-AM and higher platelet reactivity after CLOP treatment (Jiang et al., 2015). Some diseases, such as diabetes (DM) and obesity, are often associated with CLOP resistance. DM patients often suffered from reduced CLOP-mediated antiplatelet effect (Angiolillo et al., 2005; Singla et al., 2009; Mangiacapra et al., 2010; Angiolillo et al., 2011b; Angiolillo et al., 2014), which is partially due to the low plasma exposure of CLOP-AM (Angiolillo et al., 2014). DM patients also exhibited platelet abnormalities and significantly higher P2Y12 platelet reactivity (Rollini et al., 2013). Several studies have demonstrated that the platelet response to chemical stimulators in CAD patients is also less than that in healthy individuals (Peace et al., 2008; Dunne et al., 2016). All these may lead to CLOP resistance.

Physiologically-based pharmacokinetic-pharmacodynamic model (PBPK-PD model) is a feasible tool to quantitatively describe the pharmacokinetics and pharmacodynamics of drug and its metabolites. Several PBPK or PK-PD models have been used to characterize pharmacokinetic behaviors of CLOP or/and its anti-platelet effect (Yun et al., 2014; Djebli et al., 2015). For example, Djebli et al. (2015) used a PBPK model to describe pharmacokinetics of CLOP and CLOP-AM in healthy individuals carrying four CYP2C19 phenotypes after 300 mg loading dose of CLOP followed by 75 mg maintenance dose. Yun et al. (2014) developed a semi-mechanistic PK/PD model to describe the relationship between plasma concentrations of CLOP-AM and its pharmacodynamic effects . Moreover, serval studies have attempted to illustrate the effects of some genetic and demographic factors on the CLOP response in healthy individuals with population PK-PD models (Jiang et al., 2016; Samant et al., 2017).

The aim of the study was: 1) to develop a whole body PBPK-PD model characterizing CYP2C19 phenotypes to simultaneously describe concentration-time profiles of CLOP and CLOP-AM as well as its pharmacodynamic effect (indexed as inhibition of platelet aggregation, IPA) following single or multiple dose of CLOP to heathy individuals; 2) to scale the developed PBPK-PD model to CAD patients with or without DM; 3) to investigate effects of some factors such as CYP2C19 activity, CES1 activity, gastrointestinal transit rates or platelets response to CLOP-AM on plasma exposure of CLOP-AM and its IPA following oral dose of CLOP to human. The results might highlight the relationships among CLOP-AM concentrations, its IPA, CYP2C19 phenotypes and CAD with or without DM, providing a rational guidance of CLOP dose adjustment for CAD patients with or without DM.

## Materials and Methods

### Development of the PBPK-PD Model in Health Individuals

**FIGURE 1:**
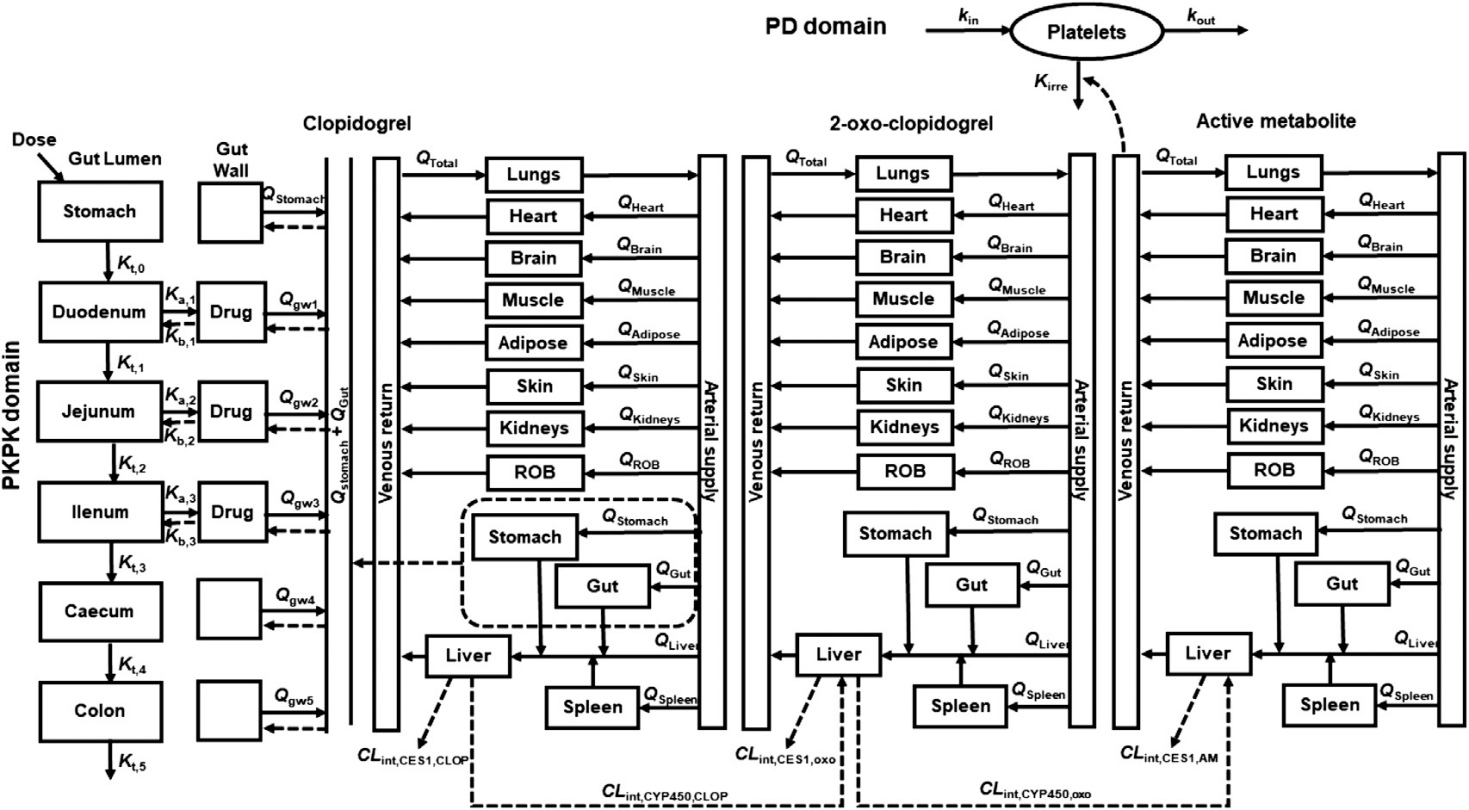
Schematic structure of whole PBPK-PD model of CLOP. Where, gw, ROB, *Q* and *Q*_total_ were gut wall compartment, rest of body compartment, blood flow rates and cardiac output. *K*_t,i_, *K*_a_, and *K*_b_ represent the gastrointestinal transit rate constants, drug absorption rate constant and drug efflux rate constant, respectively. *CL*_int,CYP450_ and *CL*_int,CES1_ represent the CYP450 and CES1-mediated intrinsic clearances respectively. *k*_irre_, *k*_in_, and *k*_out_ were CLOP-AM-mediated irreversible antiplatelet aggregation rate constant, platelet aggregation rate constant and platelet disaggregation rate constant, respectively.

A whole PBPK-PD model (Figure 1) was constructed to describe the pharmacokinetics and pharmacodynamics of CLOP and its metabolites in healthy subjects. The developed PBPK model consisted of 14 compartments: stomach, gut, lungs, heart, spleens, liver, kidneys, brain, adipose, muscle, skin, arterial blood, venous blood and the rest of body (ROB). Gut compartment consisted of gut lumen and gut wall compartments, and each of them was further divided into duodenum, jejunum, ileum, cecum and colon.

In general tissue (t):

$$V_{t}\frac{dC_{t}}{\mathrm{dt}}=Q_{t}\times \left(C_{art}-\frac{C_{t}}{K_{t/b}}\right)$$ (1)

Where *V*_*t*_, *Q*_*t*_, *C*_*t*_ and *C*_*art*_ represented the volume, blood flow rate of tissues, drug concentration in the tissues and drug concentration in artery blood, respectively. The physiological parameters used in the developed PBPK-PD model were listed in Table 1. *K*_t/b_ represented the ratio of the drug concentration in tissues to blood, which equaled to the product of ratio of drug concentration in tissue to plasma (*K*_t/p_) and ratio of drug concentration in blood to plasma (*R*_bp_), i.e. *K*_t/b_ = *K*_t/p_/*R*_bp_. The *K*_t/p_ values were estimated using method previously reported (Schmitt, 2008). The physicochemical parameters and *K*_t/p_ values of CLOP and its metabolites were listed in Table 2.

**TABLE 1:** Physiological parameters used in PBPK-PD model.

	Health		CAD	CAD + DM
	Volume (L)^a^	Blood flow (L/min)^a^	Blood flow (L/min)^b^	Blood flow (L/min)^b^
Spleen	0.16	0.16	0.14	0.14
Liver	1.38	0.42	0.38	0.38
Adipose	22.20	0.46	0.41	0.41
Muscle	17.51	0.54	0.49	0.49
Lung	0.94	5.27	4.74	4.74
Kidney	0.23	0.89	0.80	0.80
Brain	1.53	0.80	0.72	0.72
Heart	0.27	0.20	0.18	0.18
Skin	1.65	0.21	0.19	0.19
ROB ^c^	17.75	0.68	0.61	0.61
Vein	1.91	5.27	4.74	4.74
Artery	3.83	5.27	4.74	4.74
Stomach ^d^	0.15	0.13	0.12	0.12
Duodenum ^d^	0.02	0.08	0.07	0.07
Jejunum ^d^	0.06	0.30	0.27	0.27
Ileum ^d^	0.04	0.17	0.15	0.15
Caecum ^d^	0.04	0.03	0.03	0.03
Colon ^d^	0.34	0.20	0.18	0.18

a Values were cited from (Li et al., 2012).

b Values were modulated according to the calculated ratio of cardiac output in CHD patients to that in healthy subjects (Rerych et al., 1978).

c The volume of ROB was equal to the total body volume minus the sum of the organ volumes listed in the table, and the blood flow of ROB was equal to the cardiac output subtracted by the sum of blood flow in organs listed in the Table 1.

d Values were calculated according to reported ratio to total body volume and cardiac output (Perdaems et al., 2010).

**TABLE 2:** Physicochemical parameters and *K*_*t/p*_ of CLOP and its metabolites in the PBPK-PD model.

Physicochemical parameters	CLOP	2-oxo-CLOP	CLOP-AM
logP_o:w_	2.583^a^	2.23^b^	1.96^b^
pKa_1_	4.60^a^	3.945^b^	4.922^b^
pKa_2_	—	—	2.469^b^
*f*_up_ ^c^	0.02	0.0742	0.0791
*R*_bp_ ^c^	0.57	0.68	0.58
*f*_umic_ ^d^	0.015	0.180	—
*K*_t/p_ ^e^			
Adipose	6.634	0.151	0.098
Brain	6.675	8.784	3.544
Gut	6.586	4.866	1.811
Heart	6.065	6.864	2.805
Kidney	5.309	7.933	3.597
Liver	5.625	7.988	3.640
Lung	3.566	6.810	1.624
Muscle	6.171	9.525	2.802
Skin	5.309	1.390	0.601
Spleen	5.182	8.748	3.243
ROB (assumed)	0.001	0.001	0.001

a Values were cited from (Tornio et al., 2014).

b Values were calculated by Chemdraw 18.1 (PerkinElmer Informatics, Inc., Waltham, MA, United States).

c Values were cited from (Samant et al., 2017).

d Values were cited from (Djebli et al., 2015).

e Values were estimated using method previously reported (Schmitt, 2008).

In stomach:

$$\frac{dA_{0,CLOP}}{\mathrm{dt}}=-K_{t,0}\times A_{0,CLOP}$$ (2)

Where *K*_t,0_ and *A*_0,CLOP_ represented the gastric emptying rate constant and the amount of CLOP in the stomach. *K*_t,0_ was reported to be 4.8 h^−1^ (Kong et al., 2020).

In gut lumen:

Drug amount in the *i*th gut lumen (*A*_i_) was

$$\frac{dA_{i,CLOP}}{\mathrm{dt}}=K_{t,i-1}\times A_{i-1,CLOP}-K_{t,i}\times A_{i,CLOP}-K_{a,i,CLOP}\times A_{i,CLOP}+K_{b,i,CLOP}\times C_{gwi,CLOP}\times V_{gwi,CLOP}\times f_{ugut,CLOP}$$ (3)

Where *i* = 1, 2, 3, 4, and 5 represented the duodenum, jejunum, ileum, cecum and colon, respectively. *K*_t,i_ represented the constant of gastrointestinal transit rate for the *i*th gut lumen, which were reported to be 4.2, 1.8, 2.4, 0.18 and 0.06 h^−1^ for duodenum, jejunum, ileum, cecum and colon (Kong et al., 2020). *A*_i_, *C*_*gwi*_ and *V*_gwi_ were drug amount in gut lumen, drug concentration in gut wall and volume of gut wall, respectively. *f*_ugut_ represented free fraction in gut, whose value for CLOP was 0.02 (Djebli et al., 2015).

*K*_a,i_ and *K*_b,i_ represented the absorption rate constant of CLOP in *i*th gut lumen and efflux rate constant from the *i*th gut wall, respectively. The values of *K*_a,i_ and *K*_b,i_ can be estimated respectively using the effective permeability parameter (*P*_eff, A-B_) and *P*_eff, B-A_ (Qian et al., 2019):

$$K_{a,i}=\frac{2\times P_{eff,A-B}}{r_{i}}$$ (4)

$$K_{b,i}=\frac{2\times P_{eff,B-A}}{r_{i}}$$ (5)

Where *r*_i_ represented the radius of the *i*th region of intestine. Values of *r*_i_ for duodenum, jejunum, and ileum were 2.0, 1.63, and 1.45 cm (Guo et al., 2013), respectively. The *P*_eff_ was estimated using apparent permeability (*P*_app_) value obtained in Caco-2 cells based on Eq. 6 (Yang et al., 2007):

$$\log P_{eff}=0.4926\times \log P_{app}-0.1454$$ (6)

Since CLOP is a substrate of P-gp, the *P*_eff,B-A_ is mainly controlled by intestinal P-gp, the *P*_eff, A-B_ and *P*_eff, B-A_ values of CLOP might be estimated using *P*_app_ data in the presence and absence of P-gp inhibitor elacridar, i.e.

$$P_{app,CLOP}=P_{app,A-B}-P_{app,B-A}$$ (7)

$$P_{app,B-A}=P_{app,+Ela}-P_{app,CLOP}$$ (8)

Where *P*_app, CLOP_ and *P*_app, CLOP+Ela_ respectively represented *P*_app_ values of CLOP in Caco-2 cells with and without elacridar (1.20 μM), which were reported to be 0.675 × 10^−6^ and 0.133 × 10^−6^ cm/s (Taubert et al., 2006), respectively. The expression of P-gp in intestine was regional, a relative transporter scaling factor (*T*_sf,i_) was used to correct *P*_eff,B-A_. The *T*_sf,i_ values in duodenum, jejunum, ileum were estimated to be 0.64, 0.84, and 1 (Qian et al., 2019), respectively. The calculated *K*_a,i_ values in duodenum, jejunum and ileum were 0.21, 0.26, and 0.29 h^−1^, respectively; the calculated *K*_b,i_ values in duodenum, jejunum and ileum were 0.07, 0.12, and 0.16 h^−1^, respectively.

In gut wall (gwi):

For CLOP,

$$V_{gwi}\times \frac{dC_{gwi,CLOP}}{\mathrm{dt}}=\left(C_{art,CLOP}-\frac{C_{gwi,CLOP}}{K_{gut/b,CLOP}}\right)\times Q_{gwi,CLOP}+K_{a,i,CLOP}\times A_{i,CLOP}-K_{b,i,CLOP}\times C_{gwi,CLOP}\times V_{gwi}\times f_{ugut,CLOP}$$ (9)

For its metabolites,

$$V_{gwi}\times \frac{dC_{gwi}}{\mathrm{dt}}=\left(C_{art}-\frac{C_{gwi}}{K_{gut/b}}\right)\times Q_{gwi}$$ (10)

Where *Q*_gwi_ and *K*_gut/b_ represented blood flow rate in the *i*th gut wall and ratio of drug concentration in gut wall to blood, respectively.

In liver (liv):

For CLOP,

$$V_{liv}\times \frac{dC_{liv,CLOP}}{\mathrm{dt}}=Q_{liv}\times C_{art,CLOP}-\left(Q_{liv}+Q_{sp}+Q_{st}+\overset{5}{\underset{i=0}{\sum }}Q_{gwi}\right)\times \frac{C_{liv,CLOP}}{K_{liv/b,CLOP}}\;+Q_{st}\times \frac{C_{st,CLOP}}{K_{st/b,CLOP}}+Q_{sp}\times \frac{C_{sp,CLOP}}{K_{sp/b,CLOP}}+\overset{5}{\underset{i=0}{\sum }}\left(Q_{gwi}\times \frac{C_{gwi,CLOP}}{K_{gut/b,CLOP}}\right)-\left(\mathrm{PBSF}\times CL_{int,CYP450,CLOP}+CL_{int,CES1,CLOP}\right)\times \frac{C_{liv,CLOP}\times f_{ub,CLOP}}{K_{liv/b,CLOP}}$$ (11)

$$CL_{int,CYP450,CLOP}=\sum \frac{V_{max,CLOP}}{K_{m,CLOP}\times f_{umic,CLOP}+\frac{C_{liv,CLOP}\times f_{ub,CLOP}}{K_{liv/b,CLOP}}}$$ (12)

For 2-oxo-CLOP (oxo),

$$V_{liv}\times \frac{dC_{liv,oxo}}{\mathrm{dt}}=Q_{liv}\times C_{art,oxo}-\left(Q_{liv}+Q_{sp}+Q_{st}+\sum_{i=0}^{5}Q_{gwi}\right)\times \frac{C_{liv,oxo}}{K_{liv/b,oxo}}+Q_{st}\times \frac{C_{st,oxo}}{K_{st/b,oxo}}+Q_{sp}\times \frac{C_{sp,oxo}}{K_{sp/b,oxo}}+\sum_{i=0}^{5}\left(Q_{gwi}\times \frac{C_{gwi,oxo}}{K_{gut/b,oxo}}\right)+\mathrm{PBSF}\times CL_{int,CYP450,CLOP}\times \frac{C_{liv,CLOP}\times f_{ub,CLOP}}{K_{liv/b,CLOP}}-\left(\mathrm{PBSF}\times CL_{int,CYP450,oxo}+CL_{int,CES1,oxo}\right)\times \frac{C_{liv,oxo}\times f_{ub,oxo}}{K_{liv/b,oxo}}$$ (13)

$$CL_{int,CYP450,oxo}=\sum \frac{V_{max,oxo}}{K_{m,oxo}\times f_{umic,oxo}+\frac{C_{liv,oxo}\times f_{ub,oxo}}{K_{liv/b,oxo}}}$$ (14)

For CLOP-AM (AM),

$$V_{liv}\times \frac{dC_{liv,AM}}{\mathrm{dt}}=Q_{liv}\times C_{art,AM}-\left(Q_{liv}+Q_{sp}+Q_{st}+\sum_{i=0}^{5}Q_{gwi}\right)\times \frac{C_{liv,AM}}{K_{liv/b,AM}}+Q_{st}\times \frac{C_{st,AM}}{K_{st/b,AM}}+Q_{sp}\times \frac{C_{sp,AM}}{K_{sp/b,AM}}+\sum_{i=0}^{5}\left(Q_{gwi}\times \frac{C_{gwi,AM}}{K_{gut/b,AM}}\right)+\mathrm{PBSF}\times CL_{int,CYP450,oxo}\times \frac{C_{liv,oxo}\times f_{ub,oxo}}{K_{liv/b,oxo}}-CL_{int,CES1,AM}\times \frac{C_{liv,AM}\times f_{ub,AM}}{K_{liv/b,AM}}$$ (15)

Where sp and st meant spleen and stomach, respectively. *CL*_int,CYP450_ and *CL*_int,CES1_ represented CYP450 and CES1 mediated intrinsic clearances, respectively. The *CL*_int,CES1,CLOP_ value was estimated to be 276,650 1/h, accounting for 85% of CLOP’s total intrinsic clearance (Zahno et al., 2010); the *CL*_int,CES1,oxo_ and *CL*_int,CES1,AM_ value were reported to be 2,200 and 529 l/h, respectively (Samant et al., 2017). *V*_max_ and *K*_m_ represented the maximum metabolic rate and Michaelis-Menten constant of each CYP450 isoforms for CLOP or 2-oxo-CLOP. *PBSF* meant the amount of total hepatic microsomal protein, and its value was calculated by multiplying the total liver weight (g) and microsomal protein yield (mg protein/g liver weight) (Cubitt et al., 2011), which was equaled to 55,120 mg. *f*_umic_ was free faction in hepatic microsomes; *f*_ub_ was free faction in blood, which came from ratio of free faction in plasma (*f*_up_) to *R*_bp_. To investigate the contributions of CYP2C19 polymorphisms to CLOP’s bioactivation, CYP2C19 phenotypes were divided into: ultrarapid metabolizer (UM) (CYP2C19*1/*17 and CYP2C19*17/*17), extensive metabolizers (EM) (CYP2C19*1/*1), intermediate metabolizers (IM) (CYP2C19*1/*2, CYP2C19*2/*17 and CYP2C19*1/*3) and poor metabolizers (PM) (CYP2C19*2/*2 and CYP2C19*2/*3) (Simon et al., 2011). Metabolic parameters for CYP2C19 in EM, IM and PMs were estimated as follows. It was assumed that the drug affinities to CYP2C19 (*K*_m,CYP2C19_) were similar among CYP2C19 phenotypes and the main difference between different metabolizers is the difference in CYP2C19 activities (*V*_max,CYP2C19_). The activity of CYP2C19 in UMs was reported to be 1.58 fold of CYP2C19 in EM using omeprazole metabolism (Sim et al., 2006). Activities of CYP2C19 in IMs and PMs were reported to be 50 and 0% of CYP2C19 in EMs (Samant et al., 2017). The estimated metabolic parameters in Ums, EMs, IMs and PMs were list in Table 3.

**TABLE 3:** Metabolic parameters of CLOP and its metabolites used in PBPK-PD model.

	CLOP		2-oxo-CLOP		
	*V*_max_	*K*_m_	*V*_max_	*K*_m_	Enzyme content
	pmol/pmol P450/min	μM	pmol/pmol P450/min	μM	pmol P450/mg protein
CYP1A2^a^	2.27	1.58	—	—	52
CYP2B6^a^	7.66	2.08	2.48	1.62	11
CYP2C9^a^	—	—	0.855	18.1	73
CYP3A4^a^	—	—	3.63	27.8	155
CYP2C19(EM)^a^	7.52	1.12	9.06	12.1	14
CYP2C19(UM)^b^	11.88	1.12	14.31	12.1	14
CYP2C19(IM)^c^	3.76	1.12	4.53	12.1	14
CYP2C19(PM)^c^	0	1.12	0	12.1	14

a Values were cited from (Kazui et al., 2010).

b Activity of CYP2C19 in UM was 1.58 fold of CYP2C19 in EM using omeprazole metabolism (Sim et al., 2006).

c Activities of CYP2C19 in IM and PM were 50% and 0% of CYP2C19 in EM (Samant et al., 2017).

### PD Kinetics

The PD effect (indexed as IPA) was directly linked to plasma concentration of CLOP-AM in venous blood compartment, and characterized by an indirect response model (Jiang et al., 2016).

$$\frac{dM}{dt}=k_{in}-k_{out}\times M-k_{irre}\times C_{ven,AM}\times f_{ub,AM}\times M$$ (16)

Where *M* represented the maximal platelet aggregation (MPA) or platelet reactivity units (PRU) normalized from corresponding baseline. *k*_in_, *k*_out_, and *k*_irre_ meant the platelet aggregation rate constant, platelet disaggregation rate constant and CLOP-AM-mediated irreversible antiplatelet aggregation rate constant (which associated with platelets response to CLOP-AM). The *k*_out_ was estimated to be 0.007804 h^−1^ according to the reported platelet half-life time (3.7 days) (Abrahamsen, 1968). The *k*_in_ value was calculated according to the dynamic balance of platelet aggregation and disaggregation in the absence of drug intervention, i.e *k*_in_ = *k*_out_×*M*_0_, where *M*_*0*_ was equaled to 1. The *k*_in_ value was 0.007804 h^−1^. The *K*_irre_ value was estimated as 47.576 ml/nmol/h using IPA- time profile previously reported (Zhu et al., 2008) and Eq. 16 on Pheonix WinNonlin (Version 8.2, Pharsight Cooperation, st. Louis, Missouri).

The IPA was expressed as:

IPA(%)=(1−M)×100 (17)

### PBPK-PD Model in CAD Patients With or Without DM

The basic structure of PBPK-PD model in CAD patients with or without DM was similar to that in healthy individuals, while some physiological and metabolic parameters were adjusted according to the pathological characteristics of CAD and DM.

#### PBPK-PD Model in CAD Without DM Patients

The cardiac output (*Q*_total,CAD_) in CAD patients is often impaired, causing lower blood flow rates in tissues (*Q*_t,CAD_). Thus, the blood flow rates in CAD patients were adjusted using the equation:

$$Q_{t,CAD}=Q_{t,health}\times \frac{Q_{total,CAD}}{Q_{total,health}}$$ (18)

Where *Q*_total, health_ and *Q*_t,health_ were cardiac output and blood flow rates in tissues of healthy individuals, respectively. The ratio of *Q*_total,CAD_/*Q*_total, health_ was reported to be 0.90 (Rerych et al., 1978), and the adjusted blood flow rate were listed in Table 1.

Clinic trial showed that the platelet aggregation response to 20 μM ADP in CAD patients was lower than that in healthy individuals (Dunne et al., 2016), and the low response to ADP before CLOP treatment was associated to the poor response to CLOP (Samara et al., 2005). Similar report showed that the platelet aggregation response to ADP in CAD patients on aspirin was about 30% lower than that in healthy individuals (Peace et al., 2008). Thus, *k*_irre_ value in CAD patients was corrected to 0.7 times that in healthy individuals.

#### PBPK-PD Model in CAD Patients With DM

Studies have demonstrated that DM patients showed low plasma exposure of CLOP-AM and impaired CLOP response (Angiolillo et al., 2005; Singla et al., 2009; Mangiacapra et al., 2010; Angiolillo et al., 2011b; Angiolillo et al., 2014), which may be partly attributed to the altered activities of some hepatic enzymes in DM status (Yang and Liu, 2020). A report have shown that the activities of CYP1A2, CYP2B6, CYP2C9, CYP2C19 and CYP3A4 in DM statue are 1.23, 0.55, 1.26, 0.54, and 0.62 folds of that in healthy individuals (Gravel et al., 2019). CES1 activity in DM patients also altered to be 1.27 fold of healthy individuals (Miele et al., 2013). The changes of gastric emptying rate and intestinal transit time in DM status (Scarpello et al., 1976; Horowitz et al., 1996; Iida et al., 2000) were also taken into consideration for their effects on intestinal absorption. The gastrointestinal transit rates (*K*_t,i_) in stomach, duodenum, jejunum, ileum, caecum, and colon were adjusted to 2.31, 2.30, 0.99, 1.32, 0.20, and 0.04 h^−1^, according to previous report (Li et al., 2015). Moreover, DM patients showed higher expression of platelet P2Y12 (Hu et al., 2017) and higher platelet reactivity, leading to low response to some inhibitors of platelet aggregation, such as PGE1 (Kreutz et al., 2013). It was reported that inhibitory effects of PGE1 on ADP-induced platelet aggregation in DM patients was remarkably lower than that in non-DM patients (Kreutz et al., 2013). Here, *k*_irre_ value in DM patients was assumed to be *k*_irre_ value in CAD patients.

### Model Validation

Plasma concentrations of CLOP and CLOP-AM and its IPA following oral single dose and multidose administration of CLOP to healthy individuals carrying different CYP2C19 phenotypes were predicted on Phenix WinNolin software (Version 8.2, Pharsight Cooperation, st. Louis, Missouri) and compared with clinic observations. The peak concentration (*C*_max_) and area under the curve (*AUC*) values of predicted pharmacokinetic profiles were estimated using non-compartmental analysis and compared with corresponding observations. The predicted accuracies were assessed using fold-error, ratio of prediction to observation. If the fold-error fell within 0.5∼2.0, the prediction was considered successful (Parrott et al., 2005; Guest et al., 2011). Following validation in heathy individuals, the developed PBPK-PD model was scaled to CAD patients with or without DM.

Visual predicted check was performed to validate the method of PBPK-PD model in healthy individuals. Among various input parameters, *V*_max,CYP2C19_, *CL*_int,CES1_, *K*_t,i_ and *k*_irre_ showed inter-individual variability. The first order conditional estimation of the Lindstrom-Bates method was used in the simulation. For pharmacokinetic validation, the variances of *V*_max,CYP2C19_, *CL*_int,CES1_ and *K*_t,i_ with standard deviation of intra-individual error were estimated using four sets of observed CLOP-AM plasma concentration-time profiles in healthy subjects (Kobayashi et al., 2015; Umemura and Iwaki, 2016; Song et al., 2018; Zhang et al., 2020). For pharmacodynamic validation, the variances of *V*_max,CYP2C19_, *CL*_int,CES1_, *K*_t,i_, and *k*_irre_ were also estimated with three sets of reported IPA-time profiles in healthy individuals (Kim et al., 2008; Kobayashi et al., 2015; Kim et al., 2016). Then, the simulation and verification of the established population model, which based on 1,000 simulations, were performed on Pheonix WinNonlin (Version 8.1, Pharsight Cooperation, st. Louis, Missouri). The 5, 50, and 95th percentiles of the simulations were plotted along with the observed data for visual inspection.

### Sensitivity Analysis

Many metabolic enzymes are involved in the formation of CLOP-AM, in which *V*_max,CYP2C9_, *V*_max,CYP2C19_, *V*_max,CYP3A4_ and *CL*_int,CES1_ were reported to have significant gene polymorphism (Garcia-Martin et al., 2002; Zhu et al., 2008; Zhang and Finkelstein, 2019). The intestinal transit time also has its effect on pharmacokinetics of CLOP-AM by affecting the intestinal absorption of CLOP (Abuhelwa et al., 2017). Meanwhile, the platelets response to CLOP-AM greatly affected the IPA values. Thus, sensitivity analysis was conducted to evaluate the influences of variations in *V*_max,CYP2C9_, *V*_max,CYP2C19_, *V*_max,CYP3A4_, *CL*_int,CES1_, *K*_t,i_ and *k*_irre_ on the pharmacokinetics of CLOP-AM and its IPA.

### Collection of Data

The pharmacokinetic and pharmacodynamic data of CLOP following oral dose in healthy individuals, CAD with DM patients and CAD patients without DM carrying different CYP2C19 phenotypes were collected from publications on Pubmed. The data collection was based on the following criterions: 1) pharmacokinetic parameters (*C*_max_ or *AUC*) or pharmacokinetic profiles or pharmacodynamic data for healthy subjects or CAD patients following oral administration of CLOP were included. 2) diseases characteristics and CYP2C19 phenotypes were clearly illustrated; 3) the patients only used aspirin as co-medicine for antiplatelet therapy; 4) the pharmacokinetic and pharmacodynamic data might come from diﬀerent reports.

## Results

### Prediction and Validation of Pharmacokinetics in Healthy Subjects

**FIGURE 2:**
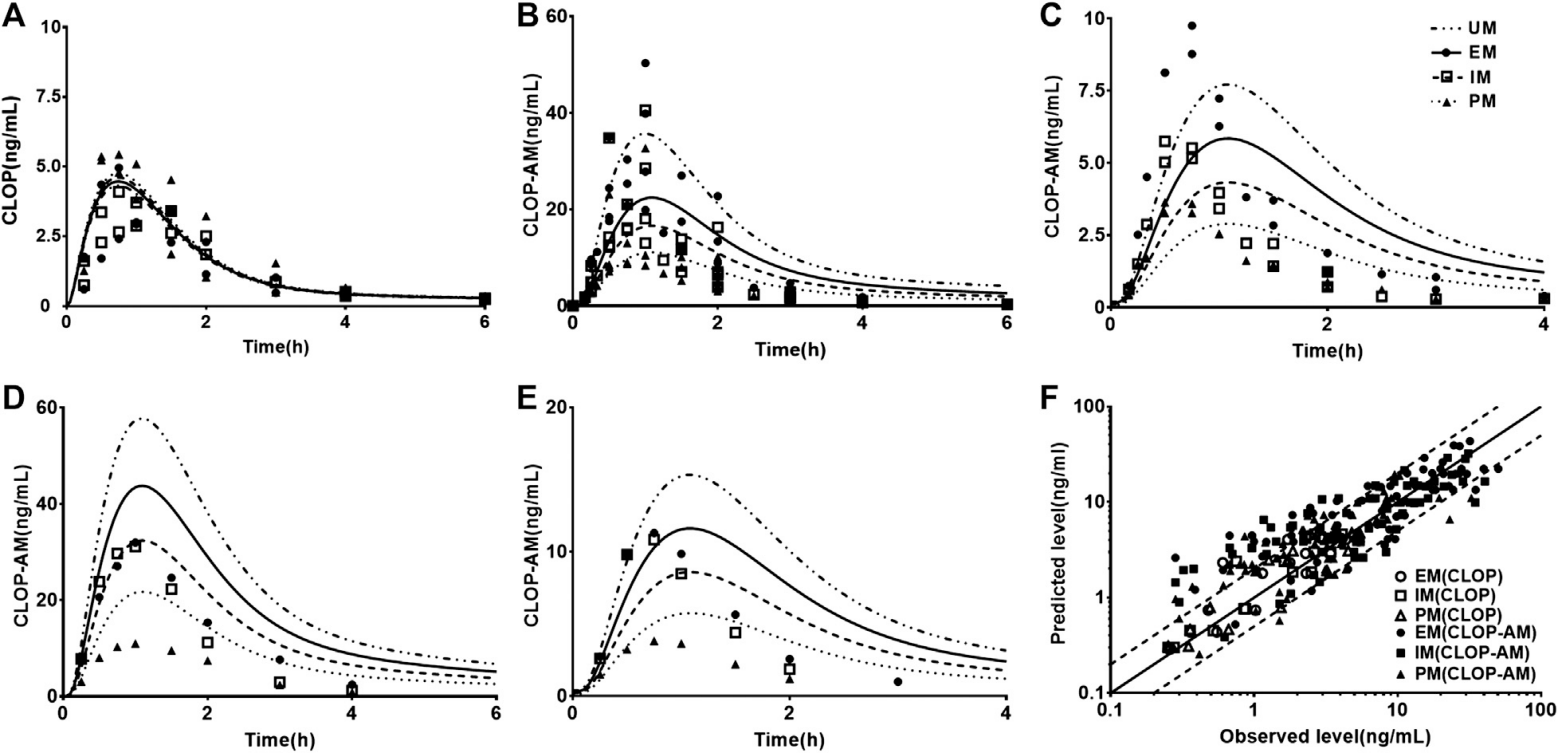
The predicted (line) and observed (point) plasma concentration-time profiles in healthy volunteers. **(A)** CLOP concentrations after 300 mg single dose. The observations were from Kim (2014, EM *n =* 7, IM *n =* 8, PM *n =* 7) (Kim et al., 2014) and Song (EM *n =* 8, IM *n =* 10, PM *n =* 2) (Song et al., 2018). **(B)** CLOP-AM concentrations after 300 mg single dose; **(C)** CLOP-AM after 300 mg loading dose followed by 75 mg maintenance dose on d7. The observations were cited came from Kobayashi (EM *n =* 9, IM *n =* 9, PM *n =* 9) (Kobayashi et al., 2015), Song (EM *n =* 8, IM *n =* 10, PM *n =* 2) (Song et al., 2018), Umemura (EM *n =* 8, IM *n =* 21, PM *n =* 7) (Umemura and Iwaki, 2016) and Zhang (EM *n =* 16, IM *n =* 16, PM *n =* 16) (Zhang et al., 2020). **(D)** CLOP-AM concentrations after 600 mg single dose; and **(E)** CLOP-AM after 600 mg loading dose followed by 150 mg maintenance dose on d7. The observed data were from Kobayashi (EM *n =* 9, IM *n =* 9, PM *n =* 9) (Kobayashi et al., 2015). **(F)** The relationship between the observed and predicted plasma concentrations of CLOP/CLOP-AM in healthy individuals. Solid and dashed lines respectively represent unity and 2-fold errors between observed and predicted data.

Fifteen sets of clinic pharmacokinetic data of CLOP and CLOP-AM following oral dose of CLOP to healthy subjects were included in the study. The plasma concentration-time profiles (Figure 2) and corresponding pharmacokinetic parameters of CLOP and CLOP-AM following different doses of CLOP (Table 4) to healthy subjects were predicted using developed model and compared with reported data (Brandt et al., 2007; Kim et al., 2008; Umemura et al., 2008; Simon et al., 2011; Kelly et al., 2012; Oh et al., 2014; Pedersen et al., 2014; Holmberg et al., 2014; Horenstein et al., 2014; Kim et al., 2014; Kobayashi et al., 2015; Kim et al., 2016; Umemura and Iwaki, 2016; Song et al., 2018; Zhang et al., 2020). The results showed that most of the predicted concentrations of CLOP-AM fell within 0.5∼2.0 folds of the observed concentrations (Figure 2F), while the predictions for pharmacokinetics of CLOP-AM after multiple doses were deviated from the clinical reports (Figures 2C,**E**). According to further data analysis, for 300 mg loading dose/75 mg maintenance doses regimen, there were 55.8% (29/52) predicted concentrations of CLOP-AM fell within 0.5∼2.0 folds of the observations (Figure 2C); and for 600 mg loading dose/150 mg maintenance doses regimen, the percentage within the acceptable range was 73.7% (14/19) (Figure 2E). It was also found that 66% (65/98) of predicted *AUC* values and 60% (57/95) of predicted C_max_ values were within 0.5–2.0 folds of clinical observations (Table 4). Moreover, among the cited 15 clinic reports, poor predictions mainly resulted from Amish reported by Horenstein (Horenstein et al., 2014) and Korean reported by Kim (Kim et al., 2014). All these results indicated successful predictions.

**TABLE 4:** The observed and predicted plasma pharmacokinetics parameters in healthy subjects.

Ref.	Race	Dose	Marker	Types	*AUC* (ng*h/ml)		*C*_*max*_ (ng/ml)	
		mg			Obs	Pre	Obs	Pre
(Horenstein et al., 2014)	Amish	75	CLOP	EM (*n =* 6)	0.61 (1,052)^a^	2.15	0.91 (348)^a^	1.12
				IM (*n =* 6)	0.54 (134)^a^	2.22	0.58 (103)^a^	1.15
				PM (*n =* 6)	0.68 (210)^a^	2.29	0.88 (99)^a^	1.19
		150	CLOP	EM (*n =* 6)	2.10 (439)^a^	4.30	1.73 (256)^a^	2.23
				IM (*n =* 6)	1.64 (378)^a^	4.44	1.30 (157)^a^	2.31
				PM (*n =* 6)	1.63 (135)^a^	4.59	1.07 (135)^a^	2.38
		300	CLOP	EM (*n =* 6)	4.91 (375)^a^	8.61	2.92 (301)^a^	4.48
				IM (*n =* 6)	1.98 (150)^a^	8.88	1.14 (301)^a^	4.62
				PM (*n =* 6)	3.50 (123)^a^	9.18	1.57 (113)^a^	4.77
		75	CLOP-AM	EM (*n =* 6)	32.7 (25)^a^	13.85	29.1 (23)^a^	5.72
				IM (*n =* 6)	33.3 (37)^a^	10.26	29.3 (30)^a^	4.23
				PM (*n =* 6)	17.4 (19)^a^	6.86	15.6 (18)^a^	2.83
		150	CLOP-AM	EM (*n =* 6)	53.6 (35)^a^	27.56	40.3 (42)^a^	11.37
				IM (*n =* 6)	43.8 (35)^a^	20.41	30.8 (45)^a^	8.41
				PM (*n =* 6)	24.2 (26)^a^	13.66	17.6 (44)^a^	5.62
		300	CLOP-AM	EM (*n =* 6)	80.4 (24)^a^	54.56	54.1 (24)^a^	22.45
				IM (*n =* 6)	73.7 (53)^a^	40.42	44.4 (71)^a^	16.61
				PM (*n =* 6)	36 (35)^a^	27.07	26.3 (44)^a^	11.11
(Zhang et al., 2020)	Chinese	300	CLOP-AM	EM (*n =* 16)	29.6^b^	54.56	22.5^b^	22.45
				IM (*n =* 16)	19.2^b^	40.42	15.1^b^	16.61
				PM (*n =* 16)	12.6^b^	27.07	8.53^b^	11.11
		300/75^c^	CLOP-AM	EM (*n =* 16)	9.38^b^	14.42	9.58^b^	5.85
		d7		IM (*n =* 16)	5.41^b^	10.69	5.59^b^	4.32
				PM (*n =* 16)	4.03^b^	7.15	3.95^b^	2.89
(Song et al., 2018)	Chinese	300	CLOP	EM (*n =* 8)	9.62 ± 3.26	8.61	3.84 ± 1.94	4.48
				IM (*n =* 10)	9.97 ± 4.31	8.88	4.90 ± 2.96	4.62
				PM (*n =* 2)	15.2 ± 0.88	9.18	7.00 ± 1.98	4.77
		300	CLOP-AM	EM (*n =* 8)	61.05 ± 21.63	54.56	45.39 ± 12.57	22.45
				IM (*n =* 10)	37.67 ± 11.01	40.42	29.15 ± 7.92	16.61
				PM (*n =* 2)	27.08 ± 2.72	27.07	19.55 ± 2.19	11.11
(Kelly et al., 2012)	Chinese	75/75^c^	CLOP-AM	EM (*n =* 34)	29.6^b^	14.42	27.6^b^	5.85
		d10		IM (*n =* 38)	21.3^b^	10.69	19.9^b^	4.32
				PM (*n =* 11)	13.9^b^	7.15	15.1^b^	2.89
(Kim et al., 2016)	Male	300	CLOP-AM	EM (*n =* 9)	51.83 ± 18.00	54.56	39.43 ± 12.00	22.45
	Korean			IM (*n =* 9)	31.27 ± 5.92	40.42	28.13 ± 10.96	16.61
				PM (*n =* 9)	15.91 ± 7.94	27.07	15.92 ± 7.59	11.11
(Kim et al., 2008)	Korean	300	CLOP	EM (*n =* 8)	10.20 ± 7.43	8.61	3.84 ± 2.5	4.48
				IM (*n =* 8)	17.02 ± 8.32	8.88	6.76 ± 3.58	4.62
				PM (*n =* 8)	29.98 ± 17.49	9.18	18.06 ± 14.26	4.77
(Kim et al., 2014)	Male	300	CLOP	EM (*n =* 7)	9.5 ± 6.4	8.61	5.5 ± 5.1	4.48
	Korean			IM (*n =* 8)	9.0 ± 7.9	8.88	4.7 ± 4.4	4.62
				PM (*n =* 7)	8.6 ± 7.8	9.18	5.5 ± 5.2	4.77
			CLOP-AM	EM (*n =* 7)	320.2 ± 107.4	54.56	152.2 ± 44.2	22.45
				IM (*n =* 8)	131.8 ± 39.7	40.42	58.3 ± 22.1	16.61
				PM (*n =* 7)	118.6 ± 40.6	27.07	64.2 ± 28.0	11.11
		300/75^c^	CLOP	EM (*n =* 7)	1.6 ± 1.3	4.78	0.8 ± 0.7	2.33
		d7		IM (*n =* 8)	2.0 ± 2.5	4.93	1.1 ± 1.7	2.40
				PM (*n =* 7)	1.6 ± 0.7	5.1	0.9 ± 0.4	2.49
			CLOP-AM	EM (*n =* 7)	87.8 ± 23.7	14.42	45.0 ± 18.6	5.85
				IM (*n =* 8)	43.3 ± 11.2	10.69	24.5 ± 8.1	4.32
				PM (*n =* 7)	30.3 ± 18.8	7.15	17.9 ± 12.5	2.89
(Oh et al., 2014)	Male	75	CLOP	EM (*n =* 9)	2.49 ± 1.62	2.15	1.77 ± 1.52	1.12
	Korean			PM (*n =* 9)	3.73 ± 3	2.29	3.09 ± 2.6	1.19
			CLOP-AM	EM (*n =* 9)	15.99 ± 4.88	13.83	14.68 ± 5.67	5.72
				PM (*n =* 9)	7.7 ± 3.22	6.86	7.17 ± 3.23	2.83
(Umemura and Iwaki, 2016)	Japanese	300	CLOP-AM	EM (*n =* 8)	104.3 ± 57.3	54.56	60.8 ± 34.3	22.45
				IM (*n =* 21)	65.5 ± 19.1	40.42	43.9 ± 14	16.61
				PM (*n =* 7)	45.1 ± 16.2	27.07	31.3 ± 13	11.11
(Kobayashi et al., 2015)	Japanese	300	CLOP-AM	EM (*n =* 9)	39.9 ± 16.8	54.56	29.8 ± 9.88	22.45
				IM (*n =* 9)	25.7 ± 6.06	40.42	19.6 ± 4.73	16.61
				PM (*n =* 9)	15.9 ± 4.73	27.07	11.4 ± 4.25	11.11
		600	CLOP-AM	EM (*n =* 9)	60.7 ± 23.4	106.96	33.3 ± 20.8	43.79
				IM (*n =* 9)	50.5 ± 21.1	79.28	32.1 ± 18.3	32.42
				PM (*n =* 9)	22.6 ± 6.95	53.18	12.0 ± 4.28	21.73
		300/75^c^	CLOP-AM	EM (*n =* 9)	11.1 ± 3.79	14.42	11.1 ± 4.67	5.85
				IM (*n =* 9)	7.20 ± 1.93	10.69	7.00 ± 3.81	4.32
		d7		PM (*n =* 9)	4.58 ± 1.61	7.15	3.90 ± 1.36	2.89
		600/150^c^	CLOP-AM	EM (*n =* 9)	15.1 ± 4.84	28.70	12.3 ± 6.34	11.62
		d7		IM (*n =* 9)	13.4 ± 4.18	21.27	11.4 ± 6.11	8.60
				PM (*n =* 9)	5.63 ± 1.28	14.24	4.42 ± 1.66	5.74
(Umemura et al., 2008)	Japanese	300	CLOP-AM	EM (*n =* 18)	58.3 ± 21.0	54.56	39.0 ± 15.0	22.45
				IM (*n =* 20)	41.5 ± 15.8	40.42	26.3 ± 11.0	16.61
				PM (*n =* 9)	33.0 ± 5.9	27.07	23.7 ± 5.9	11.11
(Holmberg et al., 2014)	Caucasian	600	CLOP-AM	EM (*n =* 7)	97.7 (65–165)^e^	106.96	NA	43.79
				IM (*n =* 5)	96.2 (45–171)^e^	79.28	NA	32.42
				PM (*n =* 2)	(77,70)	53.18	NA	21.73
(Brandt et al., 2007)	Caucasian	300	CLOP-AM	EM (*n =* 66)	76.2 ± 17.9^d^	54.56	58.4 ± 9.2^d^	22.45
				IM (*n =* 22)	41.5 ± 5.7^d^	40.42	35.3 ± 4.3^d^	16.61
				PM (*n =* 1)	26.9 (*n =* 1)	27.07	27.9 (*n =* 1)	11.11
(Pedersen et al., 2014)	Caucasian	600	CLOP-AM	UM^f^ (*n =* 11)	105.2 (62.3–166.8)^e^	140.92	71 (43–107)^e^	57.79
				UM^g^ (*n =* 9)	97.4 (52.0–183.3)^e^	140.92	69 (31–172)^e^	57.79
				EM (*n =* 11)	82.6 (51.6–123.4)^e^	106.96	64 (38–91)^e^	43.79
(Simon et al., 2011)	Caucasian	300	CLOP-AM	UM (*n =* 10)	33.9 ± 11.1	71.88	24.1 ± 9.86	29.63
	Asian			EM (*n =* 10)	39.8 ± 24.4	54.56	31.6 ± 20.6	22.45
				IM (*n =* 10)	33.6 ± 13.1	40.42	23.0 ± 10.9	16.61
				PM (*n =* 10)	16.0 ± 6.20	27.07	11.2 ± 4.0	11.11
		600	CLOP-AM	UM (*n =* 10)	56.5 ± 22.0	140.92	36.2 ± 13.4	57.79
				EM (*n =* 10)	70.6 ± 45.7	106.96	44.2 ± 27.2	43.79
				IM (*n =* 10)	56.4 ± 27.5	79.28	39.3 ± 22.5	32.42
				PM (*n =* 10)	24.4 ± 6.79	53.18	17.3 ± 5.74	21.73
		300/75^c^	CLOP-AM	UM (*n =* 10)	10.7 ± 4.52	18.99	11.7 ± 5.75	7.71
		d5		EM (*n =* 10)	11.6 ± 5.81	14.42	13.0 ± 7.33	5.85
				IM (*n =* 10)	9.87 ± 4.42	10.69	11.6 ± 5.38	4.32
				PM (*n =* 10)	3.23 ± 1.31	7.15	3.93 ± 1.39	2.89
		600/150^c^	CLOP-AM	UM (*n =* 10)	17.6 ± 7.55	37.78	15.7 ± 8.63	15.32
		d5		EM (*n =* 10)	19.3 ± 8.33	28.70	19.0 ± 4.57	11.62
				IM (*n =* 10)	16.4 ± 6.55	21.27	17.5 ± 7.12	8.60
				PM (*n =* 10)	6.79 ± 1.51	14.24	6.81 ± 1.81	5.74

a Mean (CV%).

b Least squares geometric mean.

c Loading dose/maintenance dose.

d Mean ± SE.

e Mean (range).

f CYP2C19*1/17.

g CYP2C19*17/*17. NA, no data reported.

**TABLE 5:** The observed and predicted plasma pharmacokinetics parameters in CAD patients.

Ref.	Dose	Marker	Types	*AUC*(ng.h/ml)		*C*_*max*_ (ng/ml)	
	mg			Obs	Pre	Obs	Pre
(Karazniewicz-Lada et al., 2014)	75 MD d8	CLOP	UM (*n =* 18)	5.8 ± 4.4	2.25	2.2 ± 1.6	1.1
			EM (*n =* 16)	4.2 ± 3.3	2.33	2.4 ± 2.8	1.14
			IM (*n =* 10)	4.4 ± 3.3	2.41	1.0 ± 0.6	1.18
		CLOP-AM	UM (*n =* 18)	14.4 ± 13.4	19.08	9.3 ± 7.4	7.80
			EM (*n =* 16)	14.8 ± 12 .8	14.49	8.4 ± 7.6	5.92
			IM (*n =* 10)	4.7 ± 2.3	10.74	3.0 ± 2.0	4.38
(Fallah et al., 2016)	75 MD d6	CLOP	EM (*n =* 5)	3.10 ± 2.0	2.33	1.4 ± 0.4	1.14
(Hulot et al., 2011)	300	CLOP-AM	EM (*n =* 55)	16.5 (11.2–26.1)^a^	55.14	9.0 (5.7–13.9)^a^	22.80
			IM (*n =* 41)	11.5 (8.9–17.7)^a^	40.86	7.9 (4.2–12.1)^a^	16.88
			PM (*n =* 7)	9.3 (7.6–11.2)^a^	27.40	5.1 (3.4–6.7)^a^	11.29
	900	CLOP-AM	EM (*n =* 55)	33.8 (22.2–55.8)^a^	159.03	17.3 (10.9–32.1)_a_	65.1
			IM (*n =* 41)	25 (16.9–38.1)^a^	117.96	12.7 (8.4–23.8)^a^	48.23
			PM (*n =* 7)	16.1 (11.8–18.5)^a^	79.27	6.3 (5.2–10.0)^a^	32.41
(Collet et al., 2011)	300	CLOP-AM	EM (*n =* 58)	19.60 ± 11.99	55.14	NA	22.80
			IM (*n =* 41)	14.56 ± 9.34	40.86	NA	16.88
			PM (*n =* 7)	8.71 ± 2.17	27.40	NA	11.29
	900	CLOP-AM	EM (*n =* 58)	41.62 ± 26.35	159.03	NA	65.1
			IM (*n =* 41)	31.73 ± 21.69	117.96	NA	48.23
			PM (*n =* 7)	18.09 ± 6.80	79.27	NA	32.41

a Median (range). NA, no data reported; MD, maintenance dose.

### Prediction and Validation of Pharmacodynamics in Healthy Subjects

**FIGURE 3:**
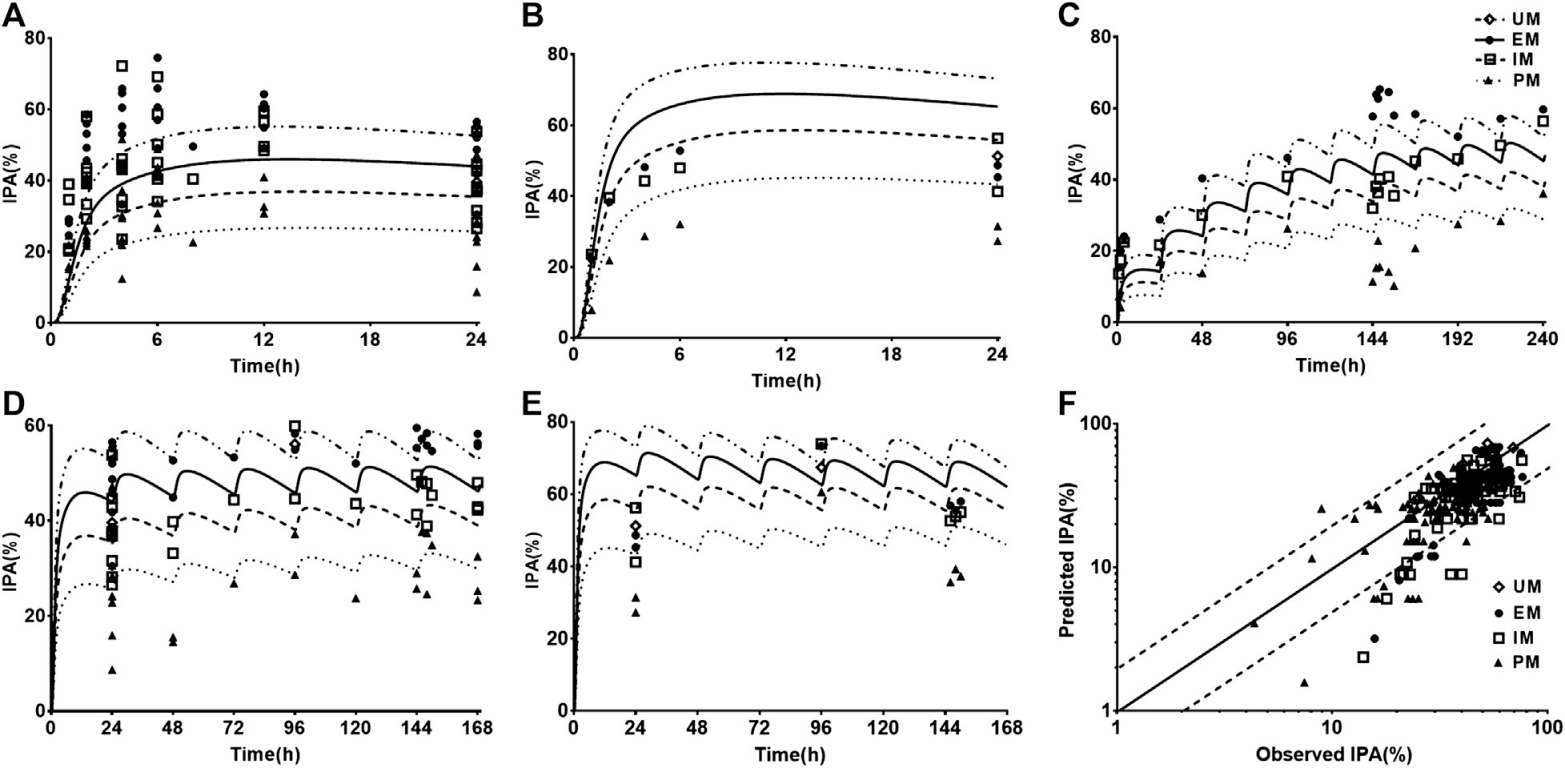
The predicted (line) and observed (point) IPA-time profiles in healthy volunteers. **(A)** IPA (%) after 300 mg single dose. The observations were cited from Kim (2008, EM *n =* 8, IM *n =* 8, PM *n =* 8) (Kim et al., 2008), Kim (2016, EM *n =* 9, IM *n =* 9, PM *n =* 9) (Kim et al., 2016), Kobayashi (EM *n =* 9, IM *n =* 9, PM *n =* 9) (Kobayashi et al., 2015), Simon (UM *n =* 10, EM *n =* 10, IM *n =* 10, PM *n =* 10) (Simon et al., 2011), Song (EM *n =* 8, IM *n =* 10, PM *n =* 2) (Song et al., 2018), Tazaki (EM *n =* 13, IM *n =* 10, PM *n =* 4) (Tazaki et al., 2012), Umemura (EM *n =* 8, IM *n =* 21, PM *n =* 7) (Umemura and Iwaki, 2016) and Zhang (EM *n =* 16, IM *n =* 16, PM *n =* 16) (Zhang et al., 2020). **(B)** IPA (%) after 600 mg single dose, whose observed data were from Kobayashi (EM *n =* 9, IM *n =* 9, PM *n =* 9) (Kobayashi et al., 2015) and Simon (UM *n =* 10, EM *n =* 10, IM *n =* 10, PM *n =* 10) (Simon et al., 2011). **(C)** IPA(%) after multidose of 75 mg, whose observations were from Horenstein (EM *n =* 6, IM *n =* 6, PM *n =* 6) (Horenstein et al., 2014), Kelly (EM *n =* 34, IM *n =* 38, PM *n =* 11) (Kelly et al., 2012) and Nakkam (EM *n =* 13, IM *n =* 18, PM *n =* 4) (Nakkam et al., 2015). **(D)** IPA (%) after 300 mg loading dose followed by 75 mg maintenance dose, whose observations were from Kim (2008, EM *n =* 8, IM *n =* 8, PM *n =* 8) (Kim et al., 2008), Kim (2014, EM *n =* 33, IM *n =* 37, PM *n =* 32) (Kim et al., 2014), Kim (2016, EM *n =* 9, IM *n =* 9, PM *n =* 9) (Kim et al., 2016), Kobayashi (EM *n =* 9, IM *n =* 9, PM *n =* 9) (Kobayashi et al., 2015), Simon (UM *n =* 10, EM *n =* 10, IM *n =* 10, PM *n =* 10) (Simon et al., 2011), Song (EM *n =* 8, IM *n =* 10, PM *n =* 2) (Song et al., 2018), Tazaki (EM *n =* 13, IM *n =* 10, PM *n =* 4) (Tazaki et al., 2012) and Zhang (EM *n =* 16, IM *n =* 16, PM *n =* 16) (Zhang et al., 2020). **(E)** IPA (%) after 600 mg loading dose followed by 150 mg maintenance dose, whose observations were from Kobayashi (EM *n =* 9, IM *n =* 9, PM *n =* 9) (Kobayashi et al., 2015), Simon (UM *n =* 10, EM *n =* 10, IM *n =* 10, PM *n =* 10) (Simon et al., 2011). **(F)** The relationship between the observed and predicted IPA (%). Solid and dashed lines respectively represent unity and 2-fold errors between observed and predicted data.

Twelve sets of pharmacodynamic data following oral dose of CLOP to healthy subjects were included in the study. The IPA-time profiles after different doses of CLOP to healthy subjects were simultaneously predicted (Figure 3) and compared with the observations (Kim et al., 2008; Simon et al., 2011; Kelly et al., 2012; Tazaki et al., 2012; Horenstein et al., 2014; Kim et al., 2014; Kobayashi et al., 2015; Nakkam et al., 2015; Kim et al., 2016; Umemura and Iwaki, 2016; Song et al., 2018; Zhang et al., 2020). The results showed that 86.8% predicted IPA values were within 0.5–2.0 folds of observations (Figure 3F), demonstrating successful predictions of pharmacodynamic effect.

### Prediction and Validation of Pharmacokinetics and Pharmacodynamics in CAD Patients

**FIGURE 4:**
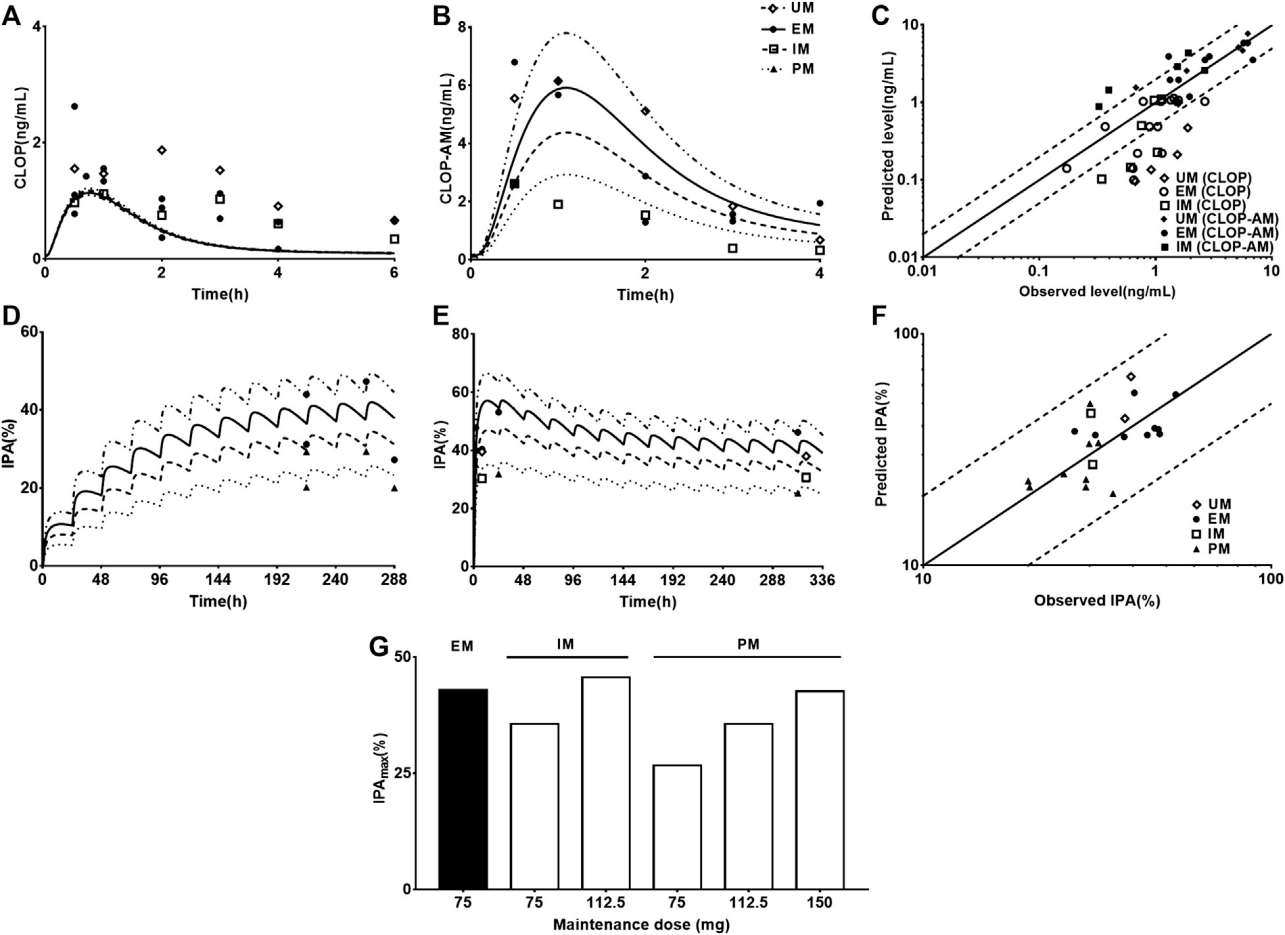
The predicted (line) and observed (point) plasma concentration-time profiles and IPA-time profiles following oral dose to CAD patients carrying different CYP2C19 phenotypes. **(A)** CLOP after 75 mg maintenance dose daily, and the observed data were from Danielak (UM *n =* 25, EM *n =* 18, IM *n =* 20) (Danielak et al., 2017), Fallah (EM *n =* 5) (Fallah et al., 2016) and Karazniewicz-Lada (UM *n =* 18, EM *n =* 16, IM *n =* 10) (Karazniewicz-Lada et al., 2014); **(B)** CLOP-AM after 75 mg maintenance dose daily, and the observed data were from Danielak (UM *n =* 25, EM *n =* 18, IM *n =* 20) (Danielak et al., 2017) and Karazniewicz-Lada (UM *n =* 18, EM *n =* 16, IM *n =* 10) (Karazniewicz-Lada et al., 2014). **(C)** shows the relationship between the observed and predicted plasma concentration of CLOP and CLOP-AM in different metabolizers. Solid and dashed lines respectively represent unity and 2-fold errors between observed and predicted data. **(D)** IPA (%) after 75 mg maintenance dose daily without loading dose, the observed data were from Erlinge (EM *n =* 49, PM *n =* 14) (Erlinge et al., 2014), Gurbel (2013, EM *n =* 36, PM *n =* 10 for smokers; EM *n =* 45, PM *n =* 9 for nonsmokers) (Gurbel et al., 2013) and Gurbel (2014, EM *n =* 153, PM *n =* 38) (Gurbel et al., 2014). **(E)** IPA (%) after 600 mg loading dose followed by 75 mg maintenance, whose observations from CHDTantry (UM *n =* 28, EM *n =* 31, IM *n =* 20, PM *n =* 3) (CHDTantry et al., 2010) and Varenhorst (EM *n =* 37, PM *n =* 9) (Varenhorst et al., 2009). **(F)** The relationship between the observed and predicted IPA (%) values in CAD patients. **(G)** The simulated dose- and CYP2C19 phenotype-dependent IPA following 300 mg loading dose followed by maintenance dose.

Following validating PBPK-PD model in heathy subjects, the developed PBPK-PD model was scaled to CAD patients. Five sets of pharmacokinetic data and pharmacodynamic data following oral dose of CLOP to CAD patient were collected in the simulations. The predicted pharmacokinetic profiles of CLOP and CLOP-AM in CAD patients received 75 mg maintenance dose daily were consistent with clinical observations (Karazniewicz-Lada et al., 2014; Fallah et al., 2016; Danielak et al., 2017) (Figures 4A,**B**), with 60% (27/45) of predicted concentrations of CLOP-AM falling within 0.5–2.0 folds of clinic reports (Figure 4C). The pharmacokinetic parameters were also estimated (Table 5). Results showed that overpredicted *C*_max_ and *AUC* values of CLOP-AM were obtained compared with data reported by Hulot (Hulot et al., 2011) and Collet (Collet et al., 2011); however, in other two sets of clinic reports, 11/14 predictions fell within 0.5–2.0 folds of observations, inferring successful predictions except for reports by Hulot and Collet. Further investigations showed that the exposure of CLOP-AM reported by Hulot (Hulot et al., 2011) and Collet (Collet et al., 2011) were remarkably lower than reports by other researchers (Karazniewicz-Lada et al., 2014; Fallah et al., 2016). IPA were further simulated with adjusted *k*_irre_ value in CAD patients and compared with clinical reports (Varenhorst et al., 2009; CHDTantry et al., 2010; Gurbel et al., 2013; Erlinge et al., 2014; Gurbel et al., 2014) (Figures 4D,**E**). The results showed that predicted IPA values were all within 0.5–2.0 folds of clinic observations (Figure 4F), demonstrating successful predictions of pharmacodynamic effect in CAD patients. Simulation analysis showed that the lower IPA in CAD patients were mainly attributed to the decrease in platelets response to CLOP-AM (expressed as *k*_irre_) and that the IPA in PM patients received 75 mg maintenance dose was only 62% of that in EM patients and that the IPA value could increase to that in EM patients when the CLOP maintenance dose for PMs increased to 150 mg (Figure 4G).

### Prediction and Validation of Pharmacokinetics and Pharmacodynamics in CAD with DM Patients

**FIGURE 5:**
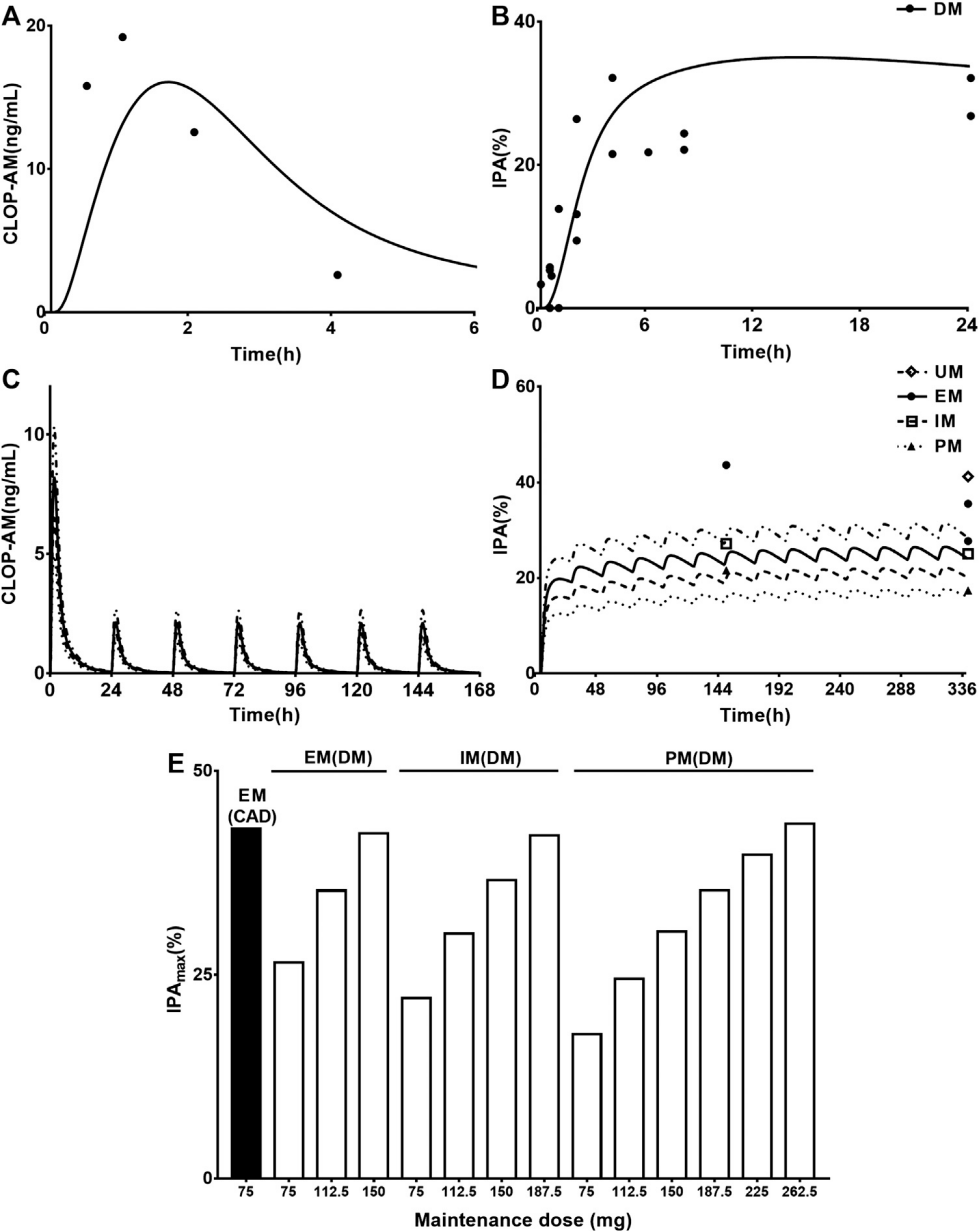
The predicted and observed pharmacokinetic and pharmacodynamic profiles in CAD patients with DM. Plasma concentration-time **(A)** profile and IPA **(B)** of CLOP-AM after 600 mg single dose to CAD patients with DM (without considering CYP2C19 phenotypes). The observations from Angiolillo (2011, *n =* 34) (Angiolillo et al., 2011a), Angiolillo (2014, *n =* 30) (Angiolillo et al., 2014), Clavijo (*n =* 21) (Clavijo et al., 2015) and Sweeny (*n =* 16) (Sweeny et al., 2017). The simulated plasma concentrations **(C)** of CLOP-AM and **(D)** IPA (%)-time profile after 300 mg loading dose followed by 75 mg maintenance dose to CAD patient with DM carrying CYP2C19 phenotypes. The observations from Carreras (EM *n =* 82, PM *n =* 27) (Carreras et al., 2016), Liu (no data) (Liu et al., 2014), Oestreich (UM *n =* 14, EM *n =* 62, IM *n =* 22) (Oestreich et al., 2014). **(E)** simulated dose- and CYP2C19 phenotype-dependent IPA following 300 mg loading dose followed by maintenance dose in CAD patients with DM, CAD-EMs without DM served as control.

One report for pharmacokinetics of CLOP-AM and four reports for IPA following oral dose of CLOP to CAD with DM patients without considering CYP2C19 phenotypes were first simulated. After adjustment of corresponding parameters, the pharmacokinetic and pharmacodynamic profiles of CLOP-AM following 600 mg CLOP single dose to DM patients were predicted (Figures 5A,B). The results showed that predicted plasma concentrations and IPA of CLOP-AM were comparable to clinic observations (Angiolillo et al., 2011a; Angiolillo et al., 2014; Clavijo et al., 2015; Sweeny et al., 2017). The predicted *AUC* and *C*_max_ were 52.46 ng*h/ml and 16.08 ng/ml, which were consistent with clinic observations (32.81 ng*h/ml and 19.77 ng/ml) (Angiolillo et al., 2014). Then, the developed PBPK-PD model was further used to simulate plasma concentrations (Figure 5C) and IPA (Figure 5D) of CLOP-AM after 300 mg loading dose followed by 75 mg maintenance dose to CAD patients with DM involving CYP2C19 phenotypes. The predicted IPA values were consistent with clinic reports (Liu et al., 2014; Oestreich et al., 2014; Carreras et al., 2016). CAD patients with DM showed lower IPA values than those in CAD without DM patients, which were in line with lower exposure of CLOP-AM, characterizing CLOP resistance. Moreover, the difference (13.7%) of IPA between PMs and UMs was also less than that (23.2%) in non-DM patients. Simulation analysis demonstrated that although the IPAs of CLOP-AM in DM patients were less than that in non-DM patients, the pharmacodynamic effect could still reach that in EM CAD patients when CLOP maintenance doses increased to 150, 187.5, and 265.5 mg for EMs, IMs and PMs of DM patients, respectively (Figure 5E).

### Visual Predictive Checks of the PBPK-PD Model in Humans

**FIGURE 6:**
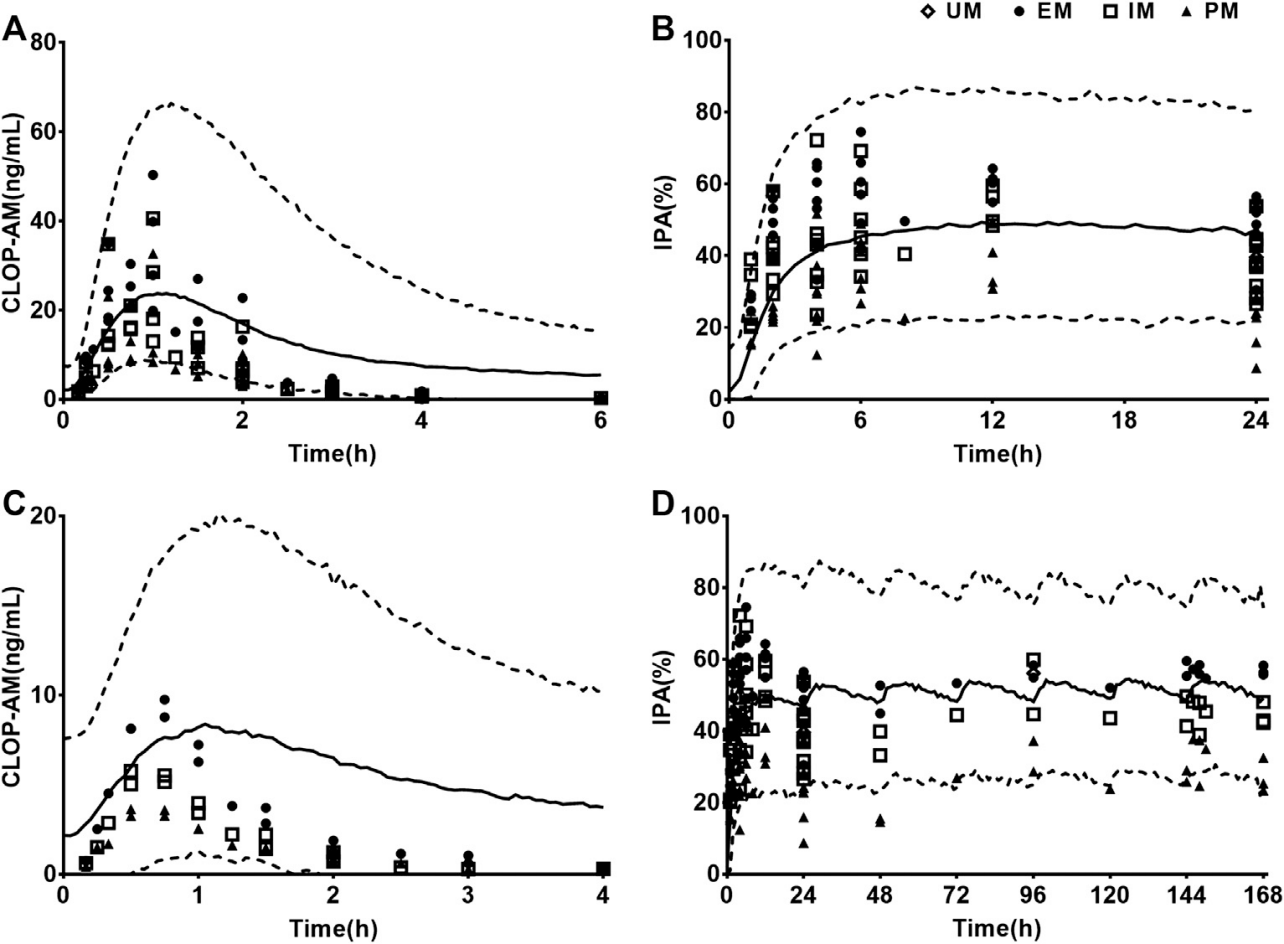
Results of visual predictive checks. Visual predictive checks of the predicted concentrations of CLOP-AM and IPA following 300 mg oral single dose of CLOP **(A,B)** or loading dose 300 mg followed by75 mg maintenance dose **(C,D)** of CLOP to healthy individuals. The observations were cited from Horenstein (EM *n =* 6, IM *n =* 6, PM *n =* 6) (Horenstein et al., 2014), Kelly (EM *n =* 34, IM *n =* 38, PM *n =* 11) (Kelly et al., 2012), Kim (2008, EM *n =* 8, IM *n =* 8, PM *n =* 8) (Kim et al., 2008), Kim (2014, EM *n =* 7, IM *n =* 8, PM *n =* 7 for pharmacokinetics; EM *n =* 33, IM *n =* 37, PM *n =* 32 for pharmacodynamics) (Kim et al., 2014), Kim (2016, EM *n =* 9, IM *n =* 9, PM *n =* 9) (Kim et al., 2016), Kobayashi (EM *n =* 9, IM *n =* 9, PM *n =* 9) (Kobayashi et al., 2015), Song (EM *n =* 8, IM *n =* 10, PM *n =* 2) (Song et al., 2018), Umemura (EM *n =* 8, IM *n =* 21, PM *n =* 7) (Umemura and Iwaki, 2016), Zhang (EM *n =* 16, IM *n =* 16, PM *n =* 16) (Zhang et al., 2020), Tazaki (EM *n =* 13, IM *n =* 10, PM *n =* 4) (Tazaki et al., 2012), Simon (UM *n =* 10, EM *n =* 10, IM *n =* 10, PM *n =* 10) (Simon et al., 2011) and Nakkam (EM *n =* 13, IM *n =* 18, PM *n =* 4) (Nakkam et al., 2015).

Visual predictive checks were performed to assess the accuracies of predictions for the plasma concentrations of CLOP-AM and IPA following oral dose of CLOP to humans (Figure 6). The observations came from different clinical observations in healthy subjects (Kim et al., 2008; Simon et al., 2011; Kelly et al., 2012; Tazaki et al., 2012; Horenstein et al., 2014; Kim et al., 2014; Kobayashi et al., 2015; Nakkam et al., 2015; Kim et al., 2016; Umemura and Iwaki, 2016; Song et al., 2018; Zhang et al., 2020). The results showed that all of the observed CLOP-AM plasma concentrations and 93.7% of the observed IPA fell between the 5th and 95th percentiles of the simulations, indicating that the predictions of CLOP-AM pharmacokinetic behaviors and IPA in human using developed PBPK-PD model were reasonable.

### Sensitivity Analysis

**FIGURE 7:**
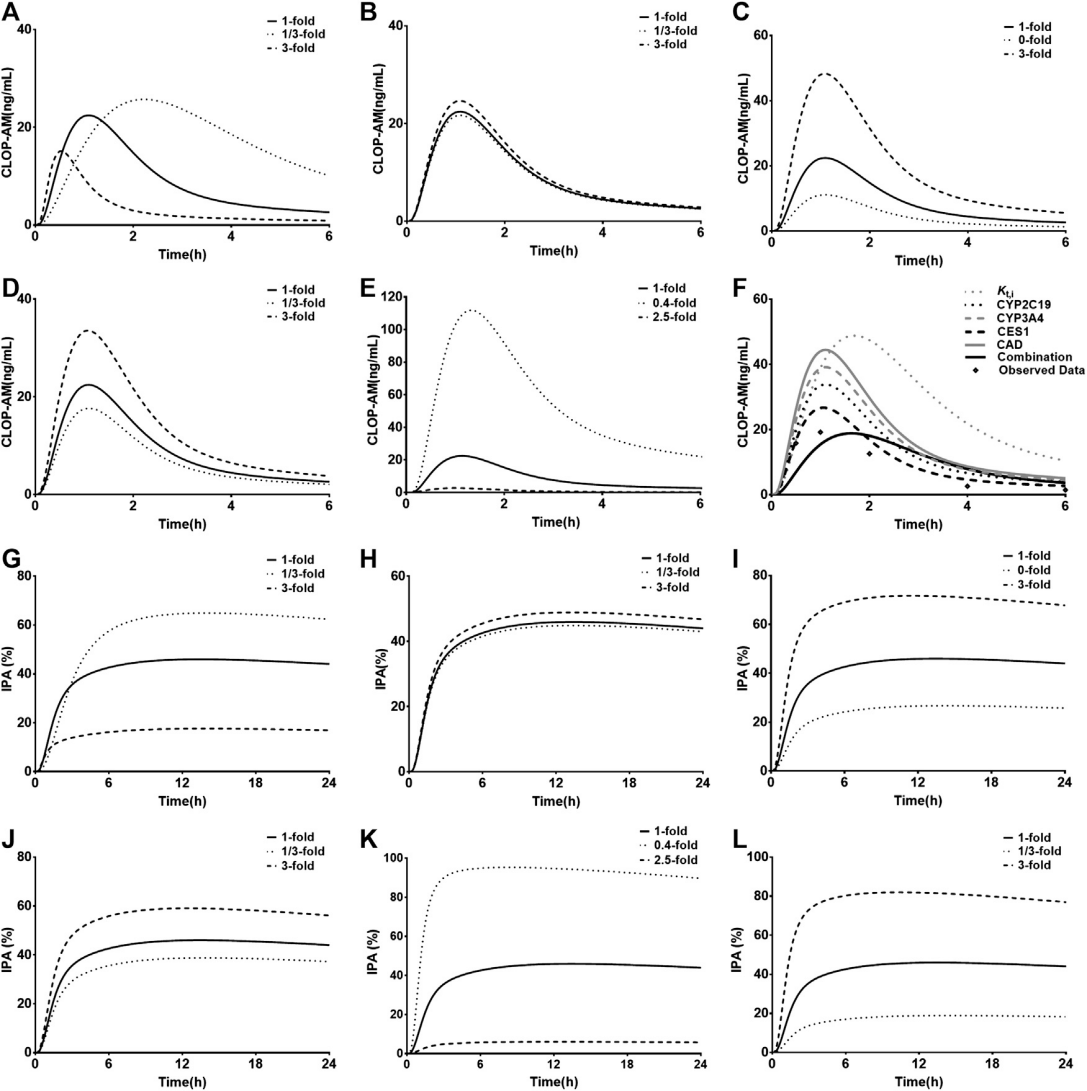
Results of sensitivity analysis. The effects of variations in *K*_t,i_ **(A)**, *V*_max,CYP2C9_ **(B)**, *V*_max,CYP2C19_ **(C)**, *V*_max,CYP3A4_ **(D)** and *CL*_int,CES1_ **(E)** on pharmacokinetic profiles of CLOP following oral 300 mg of CLOP to healthy individuals; **(F)** the individual contributions of alterations in *V*_max,CYP2C19_, *V*_max,CYP3A4_, *CL*_int,CES1_ and *K*_t,i_ to pharmacokinetics of CLOP-AM and their integrations following oral 600 mg of CLOP to CAD patients with DM. The effects of variations in *K*_t,i_ **(G)**, activities of *V*_max,CYP2C9_ **(H)**, *V*_max,CYP2C19_ **(I)**, *V*_max,CYP3A4_ **(J)**, *CL*_int,CES1_ **(K)** and *k*_irre_ **(L)** on IPA(%) of CLOP-AM following oral dose of 300 mg CLOP to healthy individuals.

*K*_t,i_, *V*_max,CYP2C9_, *V*_max,CYP2C19_, *V*_max,CYP3A4_, and *CL*_int,CES1_ (Figures 7A–E) were selected to conduct sensitivity analysis on the pharmacokinetic profiles of CLOP-AM. The results showed that the altered *V*_max,CYP2C9_ and *V*_max,CYP3A4_ values have slight effects on the pharmacokinetics of CLOP-AM, although they are involved in the formation of CLOP-AM. Increases in *K*_t,i_ and *CL*_int,CES1_ values or decrease in *V*_max,CYP2C19_ remarkably decreased the vivo exposure of CLOP-AM, and the contributions were *CL*_int,CES1_>> *K*_t,i_≈ *V*_max,CYP2C19_>> *V*_max,CYP3A4_. To mimic DM status, individual contributions of the altered *V*_max,CYP2C19_, *V*_max,CYP3A4_, and *CL*_int,CES1_ as well as *K*_t,i_ to the pharmacokinetics of CLOP-AM and their integrated contribution were investigated using CAD patients as control. The results showed that alteration in *K*_t,i_ increased the plasma exposure of CLOP-AM by 58%. In contrast, the increased *CL*_int,CES1_ (by 27%) and decreased *V*_max,CYP2C19_ (by 46%) and *V*_max,CYP3A4_ (by 38%) lowered the plasma exposures of CLOP-AM by 43, 24, and 12%, respectively. Meanwhile, their integrated effect decreased the plasma exposure of CLOP-AM (Figure 7F).

The impacts of *K*_t,i_, *V*_max,CYP2C9_, *V*_max,CYP2C19_, *V*_max,CYP3A4_, *CL*_int,CES1_, and *k*_irre_ on the IPA-time profiles of CLOP-AM were also investigated (Figures 7G–L). The results were consistent with the findings in pharmacokinetic investigation of CLOP-AM that slight alterations were observed when changing *V*_max,CYP2C9_ and *V*_max,CYP3A4_. The variations in *K*_t,i_, *V*_max,CYP2C19_, *CL*_int,CES1_ and *k*_irre_ remarkably affected the IPA, whose extents were *CL*_int,CES1_> *k*_irre_> *K*_t,i_≈ *V*_max,CYP2C19_ > *V*_max,CYP3A4_.

## Discussion

Clinical reports have demonstrated that the lower response to CLOP therapy in CAD patients with DM is attributed to the low exposure of CLOP-AM, which may be associated with the altered activities of some hepatic enzymes in DM status (Yang and Liu, 2020), such CYP2C19 and CES1. Moreover, phenomena such as platelet abnormalities, high expression of P2Y12 receptor and hypo-responsivity to chemical stimulators are also observed in DM patients (Ueno et al., 2011; Rollini et al., 2013; Hu et al., 2017). All these may lead to higher on-treatment platelet reactivity after CLOP medications in CAD patients with DM, which is related to increased risk of adverse cardiovascular events (Brar et al., 2011). Several investigators have illustrated pharmacokinetic behaviors of CLOP and CLOP-AM as well as its IPA. Djebli et al. (2015) described pharmacokinetics of CLOP and CLOP-AM following 300 mg loading dose of CLOP followed by 75 mg maintenance dose to healthy individuals carrying four CYP2C19 phenotypes. Yun et al. (2014) illustrated concentrations of CLOP-AM and its IPA using a semi-mechanistic PK/PD model. Effects of some genetic and demographic factors on the CLOP response in healthy individuals have been demonstrated using population PK-PD models (Jiang et al., 2016; Samant et al., 2017). The aim of this study was to develop a PBPK-PD model considering CYP2C19 polymorphisms to simultaneously predict the pharmacokinetics of CLOP, CLOP-AM as well as the IPA following oral administration to CAD patients with or without DM.

The developed PBPK-PD model was validated in healthy individuals. The results showed that developed model was successfully applied to predict pharmacokinetics of CLOP, CLOP-AM and IPA with most of predictions falling within 0.5–2.0 folds of observations except two clinical reports (Horenstein et al., 2014; Kim et al., 2014) (Figures 2, 3; Table 4). Visual predictive checks demonstrated that almost all the observations of CLOP-AM and its IPA fell between the 5th and 95th percentiles of the simulations, demonstrating successful predictions (Figure 6). Following validation in healthy subjects, the developed PBPK-PD model was successfully scaled to CAD patients with adjustment in blood flow rates and *k*_irre_ (Figure 4). Then the model was further scaled to predict pharmacokinetics of CLOP-AM and IPA in CAD patients with DM (Figure 5). Simulation demonstrated that, compared with non-DM patients, DM patients showed lower plasma exposures of CLOP-AM and lower IPA values. It is generally accepted that the main reason leading to CLOP resistance is CYP2C19 polymorphisms. However, sensitivity analysis showed that contributions of the indicated factors to IPA of CLOP-AM were *CL*_int,CES1_> *k*_irre_> *K*_t,i_≈ *V*_max,CYP2C19_ > *V*_max,CYP3A4_ (Figures 7G–L). Mimicked analysis also showed that contribution (24%) of decrease in *V*_max,CYP2C19_ to lower plasma exposure of CLOP-AM under DM status was less than that (43%) of increase in *CL*_int,CES1_, inferring that the decreased exposure of CLOP-AM in DM status was mainly attributed to increased CES1 activity. Moreover, diabetes also alters intestinal transit, in turn, decreasing CLOP absorption (Figure 7F). All these becoming reasons inducing CLOP resistance under diabetic status. Simulation also demonstrated that difference of IPA between UMs and PMs in DM status was 13.7%, less than that (23.2%) in non-DM patients (Figure 5E). All these might explain the fact that no significant effect of CYP2C19 genotype on platelet aggregation was observed in CAD patients with DM (Oestreich et al., 2014). Moreover, decreases in sensitivities of platelet to chemical stimulators were also reasons leading to CLOP resistance in CAD patients with or without DM. The CLOP dosage could be adjusted according to alterations in *V*_max,CYP2C19_, *CL*_int,CES1_, *k*_irre_ and *K*_t,i_ to overcome the CLOP resistance and decrease the rates of cardiovascular events under DM status (Figure 5E).

However, the model also has some limitations. For example, the two sets of predicted pharmacokinetic parameters of CLOP were not consistent with clinic observations in Amish population reported by Horenstein (Horenstein et al., 2014) and Korean population by Kim (Kim et al., 2014) (Table 4). In Korean by Kim (Kim et al., 2014), it was found that the plasma exposure of CLOP-AM following 300 or 75 mg CLOP were 5∼7 fold higher than those in other populations (Brandt et al., 2007; Umemura et al., 2008; Simon et al., 2011; Kelly et al., 2012; Holmberg et al., 2014; Horenstein et al., 2014; Pedersen et al., 2014; Kobayashi et al., 2015; Umemura and Iwaki, 2016; Song et al., 2018; Zhang et al., 2020), including other Korean population (Oh et al., 2014; Kim et al., 2016). Similarly, in Amish population reported by Horenstein (Horenstein et al., 2014), the plasma exposure of CLOP were 2–4 times lower than that in other populations (Kim et al., 2008; Kim et al., 2014; Oh et al., 2014; Song et al., 2018), and the plasma exposure of CLOP-AM were 2–4 folds times higher than those in other populations (Brandt et al., 2007; Umemura et al., 2008; Simon et al., 2011; Kelly et al., 2012; Oh et al., 2014; Kobayashi et al., 2015; Kim et al., 2016; Umemura and Iwaki, 2016; Song et al., 2018; Zhang et al., 2020). Since some factors such as sex, race, age, CES1 phenotype and the body weight were not taken into consideration in the simulations, whether the great differences between the two populations and other populations were attributed to these factors or other reasons were unclear. Meanwhile, clinic reports about CAD patients, especially CAD patients with DM considering CYP2C19 phenotypes were limited, and disease types and progression may also affect the pharmacokinetics and pharmacodynamics of CLOP-AM. The platelets response to CLOP-AM was considered to be linked to *k*_irre_, which is also affected by various factors. Here, the *k*_irre_ value in patients was assumed to be 0.7 folds of healthy individuals, whether the assumption was reasonable needed further investigation. Furthermore, the predicted plasma concentration-time profiles of CLOP-AM after multiple doses were deviated from the reported observations (Figures 2C,E), but the predicted plasma exposure to CLOP-AM and its pharmacodynamic effects were within the acceptable range according to the results of VPC (Figure 6C).

## Conclusion

The developed PBPK-PD model, which comprised altered physiological parameters, drug metabolic parameters (including CYP2C19 polymorphisms and CES1) and drug response, was successfully used to predict pharmacokinetics of CLOP-AM and its IPA in healthy individuals, CAD patients and CAD patients with DM. The model provided a feasible alternative to empirical dosage selection and guidance on dose recommendations of CLOP.

## Data Availability Statement

The original contributions presented in the study are included in the article/Supplementary Material, further inquiries can be directed to the corresponding authors.

## Author Contributions

R-jX and X-dL wrote the manuscript; J-jZ and LL designed research; R-jX and W-mK performed research; W-mK and X-fA analysed data.

## Funding

This research was funded by the National Natural Science Foundation of China, No. 81872930 and 81673505; the “Double First-Class” university project, No. CPU2018GY22.

## Conflict of Interest

The authors declare that the research was conducted in the absence of any commercial or financial relationships that could be construed as a potential conflict of interest.
